# Point-of-Care Ultrasound (POCUS) Exam Performed by an Anesthesiologist Changes Obstetric Management

**DOI:** 10.7759/cureus.78940

**Published:** 2025-02-13

**Authors:** Caitlin A Bradley, Gang Chen, Joanna Schindler, Tina S Shah, Max Schubert

**Affiliations:** 1 Anesthesiology, Emory University School of Medicine, Atlanta, USA; 2 Anesthesiology, Grady Memorial Hospital, Atlanta, USA

**Keywords:** antepartum management, heart failure with reduced ejection fraction, point-of-care ultrasound (pocus), preeclampsia with severe features, types of shock

## Abstract

Point-of-care ultrasound (POCUS) exams have been shown to impact perioperative patient care. Involvement in antepartum obstetric patients has not been previously described. We report a case of a parturient with chronic hypertension in new-onset respiratory distress. The anesthesiologist performed cardiac and lung POCUS exams that identified new-onset systolic heart failure and contributed to her diagnosis of preeclampsia with severe features. These findings guided the initiation of inotropic therapy and diuresis. The patient ultimately required transfer to a center able to perform more advanced obstetric and cardiac care, including extracorporeal membrane oxygenation (ECMO). The patient delivered via cesarean section under general anesthesia and was weaned off inotropes. This demonstrates how POCUS can guide the management of complex antepartum patients to change obstetric management.

## Introduction

Point-of-care ultrasound (POCUS) is commonly performed by anesthesiologists in the perioperative environment and has been shown to alter management and impact care [1]. For example, parturients are considered to have full stomachs with a high aspiration risk. Gastric ultrasound can provide a more accurate assessment of gastric volume, aiding in risk evaluation for parturient airways in cases where general anesthesia (GA) may be required [2]. Abdominal ultrasound performed postoperatively after cesarean section in unstable patients can rule out intra-abdominal bleeding [3]. Herein, we describe a case where lung and cardiac ultrasound significantly altered the obstetric management of an antepartum patient. Written Health Insurance Portability and Accountability Act (HIPPA) authorization from the patient was obtained for publishing this report.

## Case presentation

A 37-year-old pregnant female (G6P1213) at 33 weeks two days gestation with dichorionic diamniotic twins presented to the hospital with vaginal bleeding. She was not in labor, but imaging was concerning for a placenta previa and possible accreta from two prior cesarean sections (CS). Placenta accreta is a pregnancy complication that occurs when the placenta abnormally attaches to the uterine wall. It is part of a range of conditions called placenta accreta spectrum (PAS). The pregnancy was also complicated by severe fetal growth restriction, syphilis, herpes simplex virus, cocaine abuse, and chronic hypertension with superimposed preeclampsia with severe features. She was treated with 24 hours of intravenous magnesium (2 gm/hr) and started on nifedipine 30 milligrams daily, which was subsequently increased to 60 milligrams to improve blood pressure control. Laboratories were notable for a creatinine elevation of 1.2 milligrams/deciLiter and platelets of 128 x 10^3^/µL. Creatinine improved with hydration to 1.0 milligrams/deciLiter and platelet count rebounded to 171 x 10^3^/µL. Vitals were notable for blood pressure of 155/98 mmHg, heart rate 90 beats/min, temperature 98.4°F, respiratory rate 17 breaths/min, and oxygen saturation 99% on room air. CS was planned for 34 weeks gestation as long as there was no change in the status of the parturient or fetuses.

**Figure 1:**
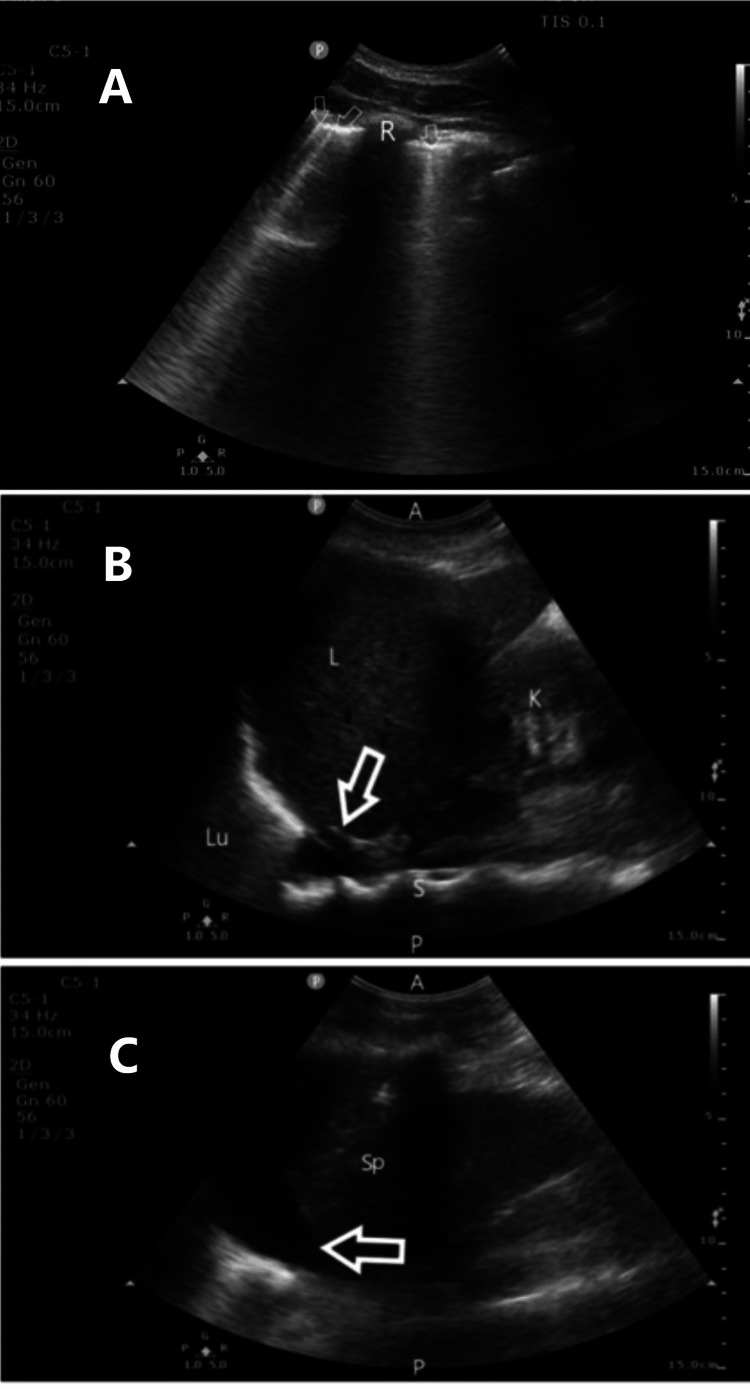
Pulmonary edema and pleural effusions Image A: Upper thorax lung field. Multiple B-lines (arrows) throughout lung fields indicating pulmonary edema. R, rib. Image B: Right-upper-quadrant ultrasound. Arrow indicates pleural effusion and clearly visible "spine sign" where thoracic spine is visible behind fluid collection. A, anterior, P, posterior, Lu, lung, S, spine, K, kidney, L, liver. Image C: Left-upper-quadrant ultrasound. Arrow indicates pleural effusion. Sp, spleen.

**Figure 2:**
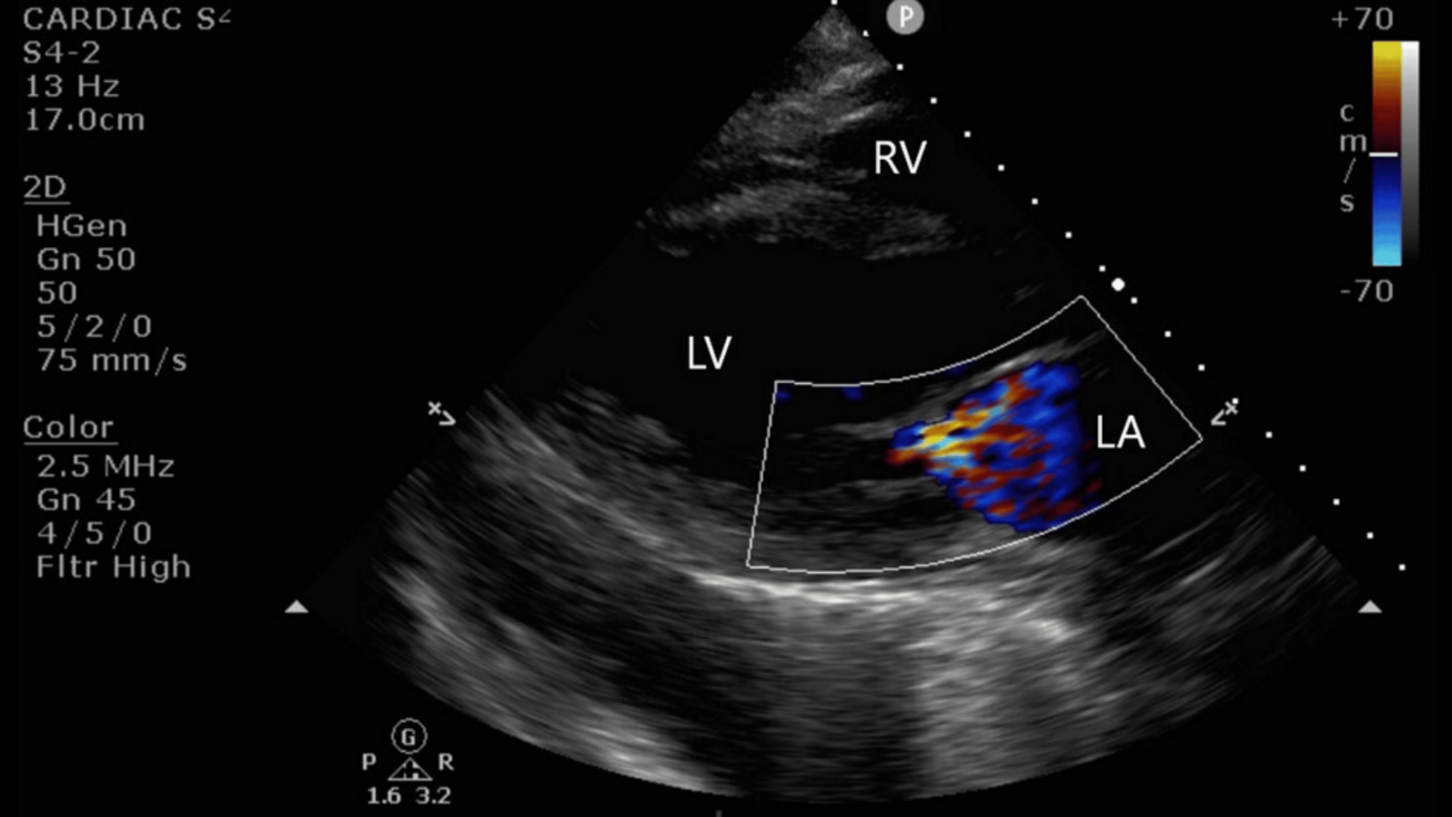
Parastenal long axis The parasternal long axis shows a thickened and dilated left ventricle (LV) and dilated left atrium (LA). Color flow Doppler (CFD) is significant for severe mitral regurgitation (MR). With CFD, severe MR is seen as a large, central jet encompassing more than 40% of the LA. An additional semiquantitative measurement for the advanced POCUS user is the vena contracta measurement. It also utilizes color flow Doppler to measure the narrowest point of the jet as it crosses the valve; greater than 0.7 cm is consistent with severe MR. RV, right ventricle.

The anesthesiologist was called by the overnight obstetric team to assist with care for the patient at 33 weeks and five days. She was in respiratory distress with an oxygen saturation of 74% on room air. A non-rebreather facemask was placed on the patient with improvement in oxygen saturation to 100%. Blood pressure was 136/60 mmHg and heart rate was 96 beats/min; fetal heart rate tracing was reassuring. The differential for sudden hypoxia was broad. Cardiac and lung POCUS were performed. Significant findings included pulmonary edema (B-lines) throughout bilateral lung fields and bilateral pleural effusions (Figure 1, Videos 1-2). Cardiac ultrasound showed moderately to severely depressed left ventricular (LV) systolic function, a dilated left atrium, and normal right ventricular systolic function (Figure 2, Video 3). The diagnosis of new-onset heart failure was made. The patient received 20 mg of intravenous furosemide and was transferred to the ICU. She was uncooperative with bilevel-positive airway pressure. New laboratories were notable for a troponin of 110 ng/L and B-type natriuretic peptide (BNP) of 1809 pg/mL. 

**Video 1:** B lines indicating pulmonary edema

**Video 2:** Bilateral pleural effusions

**Video 3:** Parasternal long axis (PLAX) The parasternal long axis (PLAX) shows a thickened and dilated left ventricle (LV) and dilated left atrium (LA). Color flow Doppler is significant for severe mitral regurgitation. RV, right ventricle. Trace pericardial and left pleural effusion are also present in the clip.

Although pulmonary status improved with diuresis, cardiac function remained unchanged on formal echocardiography, which corroborated severely reduced LV systolic function; mitral regurgitation improved to mild with diuresis and right ventricle function was preserved. Recorded POCUS images were available for comparison. Chest X-ray showed mild bibasilar opacities. In the setting of worsening renal function and rising liver enzymes, inotropic therapy was initiated with dobutamine. The patient was transferred to a high-risk center. Care was coordinated with the obstetrician, anesthesiologist, neonatologist, and cardiac surgeon. In the operating room, wires were placed in the groin to facilitate emergency extracorporeal membrane oxygenation (ECMO) cannulation in the event of cardiovascular collapse during CS. Delivery under general anesthesia was uneventful. The estimated blood loss was 850 mL. She was extubated on postoperative day one and weaned off inotropic support on postoperative day four. Postoperative transthoracic echocardiography revealed that LV systolic function remained severely depressed at <25%. LV systolic function had not recovered after post-partum cardiology appointments.

## Discussion

This case highlights the important role that anesthesiologists have in providing real-time POCUS examinations for antepartum patients. Anesthesiologists frequently interact with laboring patients who have cardiovascular diseases and hypertensive disorders of pregnancy; however, they are not frequently involved in their antepartum care. Cardiovascular disease and hypertensive disorders account for significant peripartum morbidity and mortality. Cardiovascular disease affects between 1% and 4% of pregnancies but accounts for up to 15% of peripartum mortality [4]. More women with pre-existing risk factors for heart disease, like hypertension and diabetes, are reproducing, as are women with congenital heart disease [4]. Pregnancy combined with cardiovascular disease increases the risk of complications, including exacerbation of the underlying condition, decompensation, premature delivery, and death [4]. Management of these patients is challenging when the conditions are known, but even more so when patients are late to care or have undiagnosed diseases [5]. 

Hypertensive disorders of pregnancy, which affect 2-8% of pregnancies worldwide, are also increasing in frequency [6]. A large portion of maternal mortality is attributable to hypertensive disorders, including preeclampsia [6]. Although cardiomyopathies are less common in pregnancy than other cardiovascular diseases, peripartum cardiomyopathy is a critical consideration due to its mortality rate, which ranges from 2% to 50% [4]. Peripartum cardiomyopathy develops between the last month of pregnancy to five months postpartum [4]. Dilated cardiomyopathies are more likely seen in the second trimester [4]. According to the Investigations of Pregnancy-Associated Cardiomyopathy study, a registry for peripartum cardiomyopathy outcomes, increased age, black race, multiparity, poor systolic function, and delayed diagnosis are predictors of mortality [7]. 

Signs and symptoms of heart failure in non-pregnant patients, such as peripheral edema and dyspnea on exertion, are non-specific and also seen in normal pregnancies, as well as those complicated by preeclampsia. Lung and cardiac POCUS offers anesthesiologists a reliable method for rapidly determining a diagnosis. Heart failure, including diastolic dysfunction, plays a role in the pathogenesis of pulmonary edema in parturients. Pulmonary edema can be seen with or without systolic dysfunction [8]. Diastolic dysfunction is notably an echocardiographic diagnosis. Findings include LV hypertrophy, dilated left atrium, and plethoric inferior vena cava (IVC); B-lines can also be seen with diastolic dysfunction [1]. LV systolic heart failure seen on cardiac POCUS includes reduced LV contractility (decreased thickening and excursion), LV dilation, LV hypertrophy, left atrium dilation, mitral regurgitation, and plethoric IVC. The right ventricle may also be affected by dilation and reduced contractility. 

Pleural effusions and pulmonary edema are easily seen on lung ultrasound. Lung ultrasound can be performed using any ultrasound probe: linear, curvilinear, or sector array. It is fast, reproducible, and more sensitive than a chest X-ray in the diagnosis of pleural effusion. An added benefit in this case is that ultrasound does not expose the parturient and fetus to ionizing radiation [9]. The linear probe (6-13MHz) is a higher frequency and has less penetration but is very sensitive for detecting lung sliding, the parietal and visceral pleura interface, to rule out a pneumothorax [10]. The curvilinear (5-8 MHz) and sector array probes (3-5MHz) are lower frequency and have more depth penetration [10]. As such, they can display lung sliding, evidence of pulmonary edema, and pleural effusions [10]. Normal lung tissue is not visible beyond the visceral pleura because ultrasound waves cannot be transmitted through aerated tissue. Imaging therefore relies on artifacts. Normal artifact is seen as A-lines, horizontal repetitions of the pleura caused by reverberation artifacts. Evidence of pulmonary edema, called B-lines, comet tails, or ring-down artifacts, are vertical reverberations extending from the lung parenchyma to the inferior border of the screen. The B-lines will obfuscate A-lines. Multiple anterior diffuse B-lines with lung sliding are diagnostic for pulmonary edema. Diagnosis of pulmonary edema with lung ultrasound is 97% sensitive and 95% specific when three or more B-lines are seen per lung field [10,11]. 

Pleural effusions are seen in a supine or semi-recumbent position with the curvilinear or sector array probe in the posterior axillary line. Pleural fluid is anechoic in transudative fluid and hypoechoic in exudative fluid. Because pleural fluid acts as the acoustic window, the lung border can be seen floating in fluid [10]. In mechanically ventilated patients, a fluid pocket of 5 cm suggests a drainage volume >500 mL [12]. It is estimated that multiplying 20 by the pocket depth in mm gives an estimate of drainage volume in mL [13].

A cardiac POCUS exam is performed using the sector array probe (1-5 MHz), which allows for optimal image resolution and depth penetration [10]. There are five views in a cardiac POCUS exam: parasternal long axis (PLAX), parasternal short axis (PSAX), apical four-chamber (A4C), subcostal cardiac, and subcostal IVC. A cardiac POCUS exam is designed to answer specific clinical questions, typically qualitative in nature, to help guide management. 

## Conclusions

Performing POCUS exams in a pregnant patient is another way the anesthesiologist can add value. The ability to rapidly rule in or rule out serious obstetric-specific pathologies can expedite appropriate care, a critical advantage given the high morbidity and mortality associated with cardiovascular diseases in pregnancy. As this case illustrates, the ability to use these tools in response to a change in patient status is equally valuable. The American Board of Anesthesiology incorporates cardiac and lung POCUS exams in the applied portion of board certification, emphasizing the need to include POCUS in residency training and underscoring its value in perioperative management. The ability to execute multiple types of ultrasound exams provides anesthesiologists greater insight into a broad spectrum of diseases, recognizing that pathology rarely occurs in isolation within a single organ system. The authors believe that incorporating cardiac and lung POCUS exams in obstetric anesthesia practice is an important and necessary tool for outstanding patient care.
